# Design and Validation of a Custom NGS Panel Targeting a Set of Lysosomal Storage Diseases Candidate for NBS Applications

**DOI:** 10.3390/ijms221810064

**Published:** 2021-09-17

**Authors:** Valentina La Cognata, Maria Guarnaccia, Giovanna Morello, Martino Ruggieri, Agata Polizzi, Sebastiano Cavallaro

**Affiliations:** 1 Institute for Biomedical Research and Innovation, National Research Council, 95126 Catania, Italy; valentina.lacognata@cnr.it (V.L.C.); maria.guarnaccia@cnr.it (M.G.); giovanna.morello@irib.cnr.it (G.M.); 2 Unit of Rare Diseases of the Nervous System in Childhood, Department of Clinical and Experimental Medicine, Section of Pediatrics and Child Neuropsychiatry, AOU “Policlinico”, PO “G. Rodolico”, 95123 Catania, Italy; mruggie@unict.it; 3 Department of Educational Sciences, University of Catania, 95124 Catania, Italy; agata.polizzi1@unict.it

**Keywords:** lysosomal storage disease (LSDs), newborn screening (NBS), targeted next-generation sequencing (tNGS)

## Abstract

Lysosomal storage diseases (LSDs) are a heterogeneous group of approximately 70 monogenic metabolic disorders whose diagnosis represents an arduous challenge for clinicians due to their variability in phenotype penetrance, clinical manifestations, and high allelic heterogeneity. In recent years, the approval of disease-specific therapies and the rapid emergence of novel rapid diagnostic methods has opened, for a set of selected LSDs, the possibility for inclusion in extensive national newborn screening (NBS) programs. Herein, we evaluated the clinical utility and diagnostic validity of a targeted next-generation sequencing (tNGS) panel (called NBS_LSDs), designed ad hoc to scan the coding regions of six genes (*GBA*, *GAA*, *SMPD1*, *IDUA1*, *GLA*, *GALC*) relevant for a group of LSDs candidate for inclusion in national NBS programs (MPSI, Pompe, Fabry, Krabbe, Niemann Pick A-B and Gaucher diseases). A standard group of 15 samples with previously known genetic mutations was used to test and validate the entire flowchart. Analytical accuracy, sensitivity, and specificity, as well as turnaround time and costs, were assessed. Results showed that the Ion AmpliSeq and Ion Chef System-based high-throughput NBS_LSDs tNGS panel is a fast, accurate, and cost-effective process. The introduction of this technology into routine NBS procedures as a second-tier test along with primary biochemical assays will allow facilitating the identification and management of selected LSDs and reducing diagnostic delay.

## 1. Introduction

Lysosomal storage disorders (LSDs) comprise a heterogeneous group of rare monogenic heritable (inborn) metabolism defects (~70) occurring mostly in infancy and childhood [1]. These diseases are characterized by the progressive accumulation of non-degraded substrates into the lysosomes, which leads to impaired lysosomal functions, altered metabolic processes, tissue damage, and death [1,2]. Unfortunately, there is considerable clinical variability within each disease phenotype, and also consistent overlapping symptomatology among LSDs, which makes the definition of a precise diagnosis solely based on clinical manifestations a very demanding task for clinicians [3].

The diagnostic work-up currently relies on multiple combined laboratory procedures (marking the infamous note “diagnostic odyssey”), that include either the detection of the single enzyme activity or substrates protein abundance in biological fluids, followed by biomarkers analysis and adjunct confirmatory gene sequencing tests to identify pathogenic mutations [4,5]. In this laborious and time-consuming medical process, the possibility to provide an early diagnosis plays a role of particular relevance for newborns and relative families. Firstly, a timely diagnosis means an earlier start of the treatment in order to halt disease progression and achieve a successful clinical outcome. Secondly, a timely diagnosis provides parents with realistic information about their child’s prognosis, enables appropriate genetic counseling about future pregnancies, reduces the psychological burden, and optimizes clinical management [6].

Both the evidence for the beneficial effects of the early treatment in preventing severe disabilities and death, as well as the emergence of novel rapid diagnostic methods directly based on the use of dried blood spots (DBS), has recently opened up the potential for introducing some LSDs into extended national newborn screening (NBS) programs [7,8]. Several autonomous initiatives or pilot LSDs screening programs (focusing primarily on MPSI, Pompe, Fabry, Krabbe, Niemann Pick A-B, and Gaucher diseases) are slowly spreading in a number of countries worldwide with the aim of testing the clinical utility of an early diagnosis and mainly rely on tandem mass spectrometry (MS/MS) or digital microfluidic fluorometry (DMF) technologies [6,9,10].

DNA testing for LSDs is currently used as a second-line test (adjunct test) in the NBS protocols, aiding clarification of ambiguous or borderline biochemical screening results. The searching of genetic causative variants is mainly entrusted to Sanger sequencing or qPCR systems, where the exon-by-exon or variant-by-variant approach slows down diagnostic times. Fortunately, the advent of next-generation sequencing (NGS) is revolutionizing the field of both diagnosis and screening, playing a relevant role as a genetic support tool in the diagnosis of LSDs always in combination with biochemical and clinical data [11]. With NGS, the simultaneous analysis of large numbers of genetic loci and samples can drive down costs and turnaround time; laboratory processes can largely be automated, and a single assay can be used to screen a set of disorders (regardless of whether a biochemical marker is available) [12,13].

In the present study, we aimed to design and evaluate both the clinical and diagnostic validity of a semi-automated and comprehensive sequencing assay based on a targeted NGS (tNGS) panel (hereafter referred to as NBS_LSDs) to screen variants in six genes (*GBA*, *GAA*, *SMPD1*, *IDUA1*, *GLA*, *GALC*) whose mutations are responsible for a set of LSDs (MPSI, Pompe, Krabbe, Fabry, Gaucher, and Niemann Pick A-B diseases) that are candidates for inclusion in NBS programs. We also assessed time and costs in order to estimate the opportunity for National Health Systems to introduce this technology in routine screening programs.

## 2. Results

### 2.1. Performance and Coverage Analysis of the NBS_LSDs Ion AmpliSeq Panel

The NBS_LSDs panel was designed to target both the entire coding regions of six LSDs-related genes (*GBA*, *GAA*, *SMPD1*, *IDUA1*, *GLA*, *GALC)* and their relative 5′ and 3′ UTRs with an exon padding of at least 10 bp on either side of each exon. The panel included 157 amplicons (with a length of 230–275 bp) distributed between two primer pools (79 + 78 primer pairs) and covered a size of 29.28 kb (the complete design of NBS_LSDs panel is available in Supplementary file S1). No additional intronic regions were targeted to maximize the coverage of exonic regions and to facilitate rapid and unambiguous interpretation in the context of NBS. To assess the efficiency and accuracy of the panel, we used a reference group of standard DNA samples isolated from clinically diagnosed donor subjects (*n* = 15, including 4 Gaucher disease, 3 Fabry disease, 3 Pompe, 3 Niemann Pick A-B, 2 MPSI) obtained from the NIGMS Human Genetic Cell Repository at the Coriell Institute for Medical Research. We used the Ion Chef System and the Ion Torrent S5 Gene Studio platforms since the rapid automated amplicon-based library preparation enables a fast turnaround time without excessive operator efforts and affords a high degree of sample multiplexing and throughput. Up to 32 barcoded libraries (8 samples pool for each Chef run) were super-pooled in equimolar concentration (40 pM each) and processed through high throughput sequencing.

**Figure 1:**
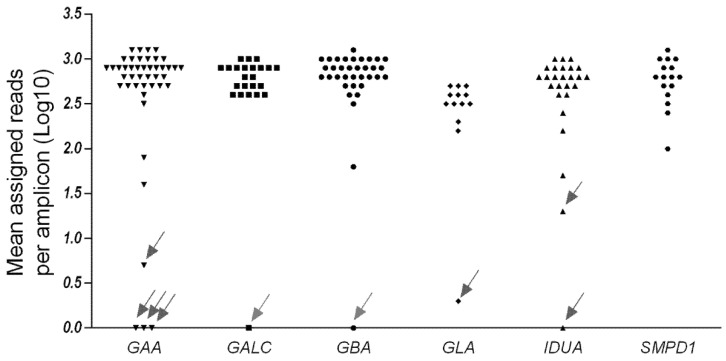
Amplicon coverage of target genes. A total of 157 amplicons distributed across six genes were amplified and sequenced with the made-to-order Ion AmpliSeq NBS_LSDs panel. This chart shows the mean coverage of individually targeted amplicons across each gene for 15 standard samples. The arrows indicate the amplicons with low amplification efficiency. Amplicons with zero reads were arbitrarily represented as 0 Log10.

From the run metrics results, all samples were uniformly covered at depths that exceeded the minimum coverage required (30×) for the accurate calling of variants. Coverage analysis shows that 148/157 of the amplicons (95%) had a sufficient amplification efficiency (mean assigned reads per amplicon Log10 ranging from 1.5 to 3), while 9 amplicons (1 for *GBA*, 1 for *GALC*, 4 for *GAA*, 2 for *IDUA*, 1 for *GLA*) were below the threshold (Figure 1 and Supplementary file S2). Amplicons with zero reads were arbitrarily represented as 0 Log10.

Filtering pipeline on the TVC (Torrent Variant Caller) was based on a stepwise strategy (i.e., coverage min 30×, *p*-value < 0.01, ClinVar ≠ Benign or Likely Benign, MAF < 0.001, Frequency 40–60% for heterozygous variants and >85% for homozygous variants, variants effects ≠ synonymous, include intronic variants if the distance from exon is <15 bp) to highlight relevant variants. Comparison with the previously known variants reported in the Coriell biobank was performed by post-filtering analysis. True positives (TPs), true negatives (TNs), false positive (FPs), and false negative (FNs) variant calls were defined by considering available data from the single causative gene in the Coriell repository (see the Material and Methods section).

The overall accuracy of the panel was 98.2%, analytical sensitivity was 90.9%, while specificity was 100%. There were 20 correctly called true positive variants, 91 true negative reference calls, and 2 false negative (missed) calls by comparing our results with expected variants (Table 1).

### 2.2. Case-Reports for Standard Samples: Pitfalls and Findings

The majority of detected pathogenic mutations and polymorphisms were consistent with the data reported in the Coriell biobank; however, some pitfalls and some interesting findings emerged as described below.

#### 2.2.1. Pitfalls

The NBS_LSDs panel missed detecting two expected variants in the NA00107 and NA10874 samples (false negatives). The first one, the NA00107 DNA sample, was hemizygous for a G>A change in exon 3 of the GLA gene, producing a stop codon (c.485G>A, Trp162Ter) (Table 1). This variant is localized inside the amplicon ES19_GLA8 characterized by a low amplification efficiency in all processed samples (Figure 1 and Supplementary File S2), although this amplicon was imported from a parent design from the Ion Community.

**Figure 2:**
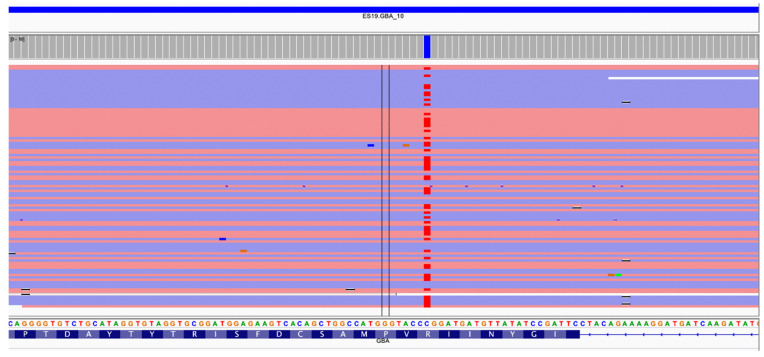
IGV (Integratice Genomics Viewer) visualization of the coverage analysis plugin for the amplicon ES19.GBA_10 covering the variant 476G>A (R120Q) for the sample NA10874. The variant is excluded by TVC because of the very low MQV (mapping quality value) of the reads due to the presence of a repeat.

The second false negative was observed in the NA10874 DNA sample. This latter is a compound heterozygote, carrying both the 1226A>G (N370S) and the 476G>A (R120Q) transitions in *GBA*. Using the NBS_LSDs panel, the diagnosis was inconclusive since the 476G>A, although partially present in raw data (Figure 2), was excluded by TVC because of the very low MQV (mapping quality value) of the reads due to the presence of a repeat (Table 1).

#### 2.2.2. New Findings

Although the LSDs are clearly recognized as monogenic diseases (mainly inherited with recessive traits), the variability of symptoms manifestations, as well as the phenotypic overlapping between genetically different disorders, make the diagnosis difficult. In order to refine the understanding of the genotype–phenotype correlations, the presence of additional secondary variants in not causative genes, but involved in lysosomal regulation and metabolism, should be considered. The use of a comprehensive sequencing panel may allow focusing on those variants that, although not pathogenic alone, may reduce enzymatic activity and contribute to phenotypic manifestations. In this context, it is noteworthy the identification of additional variants in our samples.

The NA00372 and the NA00798 DNA samples, both acquired from the Coriell biobank, were successfully assessed by our sequencing panel and bioinformatic pipeline as carriers of two heterozygous mutations in *GBA* and a single homozygous variant in *IDUA*, respectively; however, after filtering the pipeline, additional variants were observed (Table 1). In particular, in sample NA00372, we observed both an additional heterozygous exonic variant (c.56A>G) in the *SMPD1* gene, causing a missense aminoacidic change (p.Gln19Arg), and a c.2561G>A exonic missense variant in *GAA* (p.Arg854Gln) targeted by ClinVar as a conflicting interpretation of pathogenicity. In the NA00798 DNA sample, we observed an additional heterozygous likely pathogenic missense variant (c.1448T>C, p.Leu483Pro) in *GBA*.

Interestingly, the Coriell biobank reports in the NA13205 DNA sample, affected by Niemann Pick type A-B, a single deletion in the *SMPD1* gene [990delC] resulting in a frameshift leading to the formation of a premature stop (TGA) at codon 382 [P330fsX382] (Table 1). There is no mention of the heterozygosity or homozygosity state. Our results demonstrated that the described variant has a frequency of 50% (heterozygous) and that the donor subject is a compound heterozygote with the second allele carrying an exonic c.1172A>C missense mutation (p.Asn391Thr) previously associated with Niemann Pick A-B disease [14].

### 2.3. Turnaround Time, Cost for NBS_LSDs tNGS Processing, and Ease of Management

The sequencing pipeline based on Ion Torrent technology (Thermo Fisher Scientific) is a semi-automated process that could be conducted inside hospital NBS screening labs starting from the same Guthrie card and blood spot samples used for primary screening, without further sample requirement.

**Figure 3:**
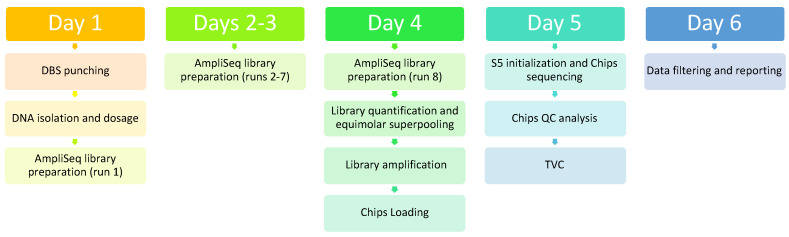
Turnaround time for processing 64 samples, from DBS (dried blood spots) punching and DNA isolation to filtering of relevant variants.

Up to 64 (4 × 8 × 2) libraries in two 510 chips (32/chip) can be loaded and sequenced simultaneously, with the entire process from collection of samples to reporting of results fitting into a six-day turnaround time. Collection of Guthrie cards, DBS punching, DNA extraction, and quantification by real-time PCR can be carried out on day 1. Eight runs of library preparation with the Chef system (for a total of 64 samples), including barcode ligation, targets amplification, and purification, can be carried from day 1 to day 4. Library quantity control, equimolar library super-pooling, and chip loading can be carried on day 4; sequencing and data processing on day 5; data analysis and reporting on day 6 (Figure 3).

Excluding the cost for personnel, plastics, and maintenance of the instruments, the cost of the entire semi-automated flowchart process, including punching of samples, DNA extraction and quantification, library preparation with Chef System, library dosage, and sequencing, is approximately 217 euro/sample (Table 2).

The estimation of both turnaround time and costs was performed considering the maximum number of samples that can be processed with the Ion Chef System and the Torrent S5 technology and using the smallest chip format (510) commercially available by Thermo Fisher Scientific. Given the rarity of these diseases, an advantage relies on the customized multiplex tNGS panel that allows collecting and processing together first-tier screen-positive samples (meaning the number of below cutoff samples measured in the first-tier NBS analysis) derived from all six evaluated pathologies. The estimation of the screen-positive rate across labs worldwide depends on multiple variants (the established cutoff value, the platform used DMF or MS/MS, and the local incidence of the disease) [9,15]; however, each lab can easily modify the proposed pipeline according to needs, meaning decide whether: (i) to completely fill and process simultaneously two chips (64 samples) or a single one (32 samples); (ii) to use the Chef System for preparing and loading libraries or to manually process samples; (iii) to enlarge the analysis to family members (parents and/or brothers). Each change at the flowchart will correspond to a variation in estimated turnaround time and costs. Nonetheless, if the proposed pipeline is maintained and a single sample is processed, the final cost would be around EUR 2300.

## 3. Discussion

Due to multiple reasons, such as wide clinical and genetic heterogeneity as well as shared clinical features, the accomplishment of a final diagnosis for LSDs constitutes a tricky challenge for clinicians [16]. The established diagnostic approach currently includes a number of medical and laboratory practices (including clinical evaluations, biochemical tests to detect the accumulated substrate or the activity of the deficient enzyme, Sanger sequencing), resulting in a delayed, expensive, and time-consuming diagnostic response [17]. Fortunately, NGS technology (in the form of WGS, WES, or tNGS panel) is becoming more accessible to the majority of labs and relatively affordable for both the diagnostic routine and NBS settings, entering in the toolbox of the diagnostic medical community. The implementation of custom-designed tNGS panels could be decisive since it allows for the simultaneous sequencing of multiple LSDs-related genes with great depth of coverage, manageable interpretation, and relatively low risk of finding variants of unknown significance, decreasing turnaround times for the final report [16,18,19].

Herein, we designed and evaluated the clinical utility of a tNGS panel (NBS_LSDs) to simultaneously screen six genes (*GBA*, *GAA*, *SMPD1*, *IDUA1*, *GLA*, *GALC*) whose mutations are responsible for a group of six LSDs (MPSI, Pompe, Krabbe, Fabry, Gaucher, and Niemann Pick A-B diseases) that are a candidate for inclusion in NBS programs. Indeed, while both MPSI and Pompe diseases have been formally recommended since 2016 for inclusion in NBS by the Recommended Uniform Screening Panel (or RUSP-a list of conditions that every baby should be screened for based on early treatment efficacy), the inclusion of Krabbe, Fabry, Gaucher, and Niemann Pick A-B diseases in few pilot NBS studies promoted by regional or local Health Government indications has been already tested (see [6]), showing both advantages and feasibility, and thus foreshadowing its extension to a larger newborn population. Noteworthy, the two main platforms currently used for the screening of LSDs (i.e., digital microfluidic fluorometry, DMF, and the tandem mass spectrometry, MS/MS, platforms) work in a multiplex assay, detecting simultaneously the six enzymes activities (i.e., DMF) or enzymatic products (i.e., MS/MS) respectively responsible for all the candidate diseases [6]. For further details about treatments and biomarkers, the reader is referred to a previous study [6].

By using a set of standard samples purchased from the Coriell Institute biobank (https://www.coriell.org/, 15 June 2020), we assessed the overall accuracy (98.2%), analytical sensitivity (90.9%), and specificity (100%) of the panel. Known pathogenic mutations in standard DNA samples were identified with the correct homozygous/heterozygous state. We also estimated the turnaround time (~6 days for 64 samples) and the cost-effectiveness for clinical sequencing (~217 euro/sample). Traditional genetic testing (Sanger sequencing) of just one of the six genes would be more expensive and time-consuming.

Several published papers have shown the possibility of carrying out successful NGS sequencing studies from DNA extracted from Guthrie card (DBS) fingerprints, thus taking advantage of the possibility of using the same non-invasive sampling from newborns for both biochemical tests and sequencing [20,21]. In order to investigate the likely response of the LSD_NBS panel from suboptimal DNA samples, we performed preliminary sequencing tests (data not shown) using a group of DNA (*n* = 5) isolated from DBS with an opportune extraction protocol (QIAamp DNA Micro kit—Isolation of Genomic DNA from Dried Blood spots—Qiagen, Hilden, Germania) and quantified with the qPCR assay as previously reported. Results showed that: (a) extracted DNA concentration was sufficient for processing samples with the LSD_NBS panel (i.e., 10 ng, the minimum amount of DNA requested for preparing libraries onto the Ion Chef System); (b) amplicons coverage was comparable to data obtained from standard samples, i.e., the same 9/157 amplicons were below the minimum average coverage (mean assigned reads per amplicon Log10 < 1.5), while the majority had a higher coverage.

Although there are some drawbacks, such as the inability to detect large indels and structural variants, the application of a tNGS-based panel (such as the one used here) as a second-tier test for NBS has the advantage of improving the performance of primary biochemical tests by reducing false positives (and parental anxiety), identifying de novo variants, and distinguishing genotypes associated with milder phenotypes. Moreover, a definitive diagnosis can be achieved earlier and at lower costs than Sanger sequencing, leading to a timely beginning of opportune therapies. Attention must be paid to primer pairs with low amplification efficiency, as well as to variant calling and interpretation. In particular, additional improvements should be oriented to (a) develop a different panel by replacing not-working amplicons, and (b) devise better algorithms to improve variant calls close to repeat regions. Nonetheless, the current design undoubtedly represents a starting point for further adjustments and improvements in order to increase the analytical sensitivity of the test.

A concerning point regards the burden of disease mutations and their likely combinations in non-pathogenic-genes, meaning the effect of cumulative mutations in lysosomal pathways that may act synergistically on phenotypes [22,23]. These data are currently not monitored by NBS or diagnostic studies because of the number of ethical and social concerns raised and represent a hot topic of discussion for the scientific and medical community. Indeed, one of the current major challenges is how to accurately interpret the clinical significance of incidental findings (variants of unknown significance, VUS, such as peri-gene sequence variants, mutations localized in intronic regions and in UTRs, or synonymous variants having an impact on gene regulation) and the scenario these uncertain data would have on patients and families as well as professional responsibilities and individual or parental choices [6]. Nonetheless, these data are crucial to reveal and refine the genotype–phenotype correlations, and the NGS-based approach is the only valid alternative for their detection, since it allows for monitoring of a broader spectrum of variants than single Sanger test, thus helping the understanding of complex cases and aiding to refine phenotype–genotype correlations. The scientific and medical community should invest in a combined effort in order to draw up informative guidelines that can properly direct clinicians and geneticists towards the right criteria for results interpretation and diagnostic report writing, but at the same time allowing advancement of collective knowledge for better management of the diseases.

## 4. Materials and Methods

### 4.1. Samples Collection and Dosage

A reference group of standard DNA samples isolated from clinically diagnosed donor subjects (*n* = 15, including 4 Gaucher disease, 3 Fabry disease, 3 Pompe, 3 Niemann Pick A-B, 2 MPSI) were obtained from the NIGMS Human Genetic Cell Repository at the Coriell Institute for Medical Research (https://www.coriell.org/, 15 June 2020—NA00372, NA00877, NA10870, NA10874, NA00107, NA00636, NA04391, NA00244, NA01935, NA14108, NA00112, NA13205, NA16193, NA00798, NA01256). Purchased samples were chosen for known variants localized in targeted genes and selected in order to ensure an adequate representation of all genes (when possible). Quantification of genomic DNA was assessed by measuring the genomic copies of the human *RNase P* gene by using the TaqMan^®^ RNase P Detection Reagents Kit (Thermo Fisher Scientific, Waltham, MA, USA) and the Aria Dx Real-Time PCR System (Agilent Technologies, Santa Clara, CA, USA).

### 4.2. Panel Design and Library Preparation

A made-to-order Ion AmpliSeq panel (IAD199968, hereafter referred to as the “NBS_LSDs” panel) was designed on the Ion AmpliSeq designer software (https://ampliseq.com, 15 May 2020, Thermo Fisher Scientific, Waltham, MA, USA) to cover all the coding regions of six genes (*GBA*, *GAA*, *SMPD1*, *IDUA1*, *GLA*, *GALC*) whose mutations are responsible for MPSI, Pompe, Krabbe, Fabry, Gaucher, and Niemann Pick A-B diseases. The panel extends 10 bp on either side of each exon and also covers the 5′ and 3′ untranslated region (UTR). When possible, amplicons were imported from Ion AmpliSeq Community Panels (used parents designs: ES_Epilepsy and ES_IEM v2).

Library preparation was carried out using the Ion AmpliSeq Kit for Chef DL8 (DNA to Library, 8 samples/run) used for automated library preparation of the Ion AmpliSeq libraries on the Ion Chef System (Thermo Fisher Scientific, Waltham, MA, USA). According to the recommended number of amplification cycles in the standard protocol, the amplification conditions were set out to 23 cycles and four minutes of annealing/extension time. At the end, library quality and molarity were assessed by using the Ion Library TaqMan^®^ Quantitation Kit (Thermo Fisher Scientific, Waltham, MA, USA) on the Aria Dx Real-Time PCR System (Agilent Technologies, Santa Clara, CA, USA). Serial dilutions of the *E. coli DH10B* Control Library were prepared and ran in triplicate to generate a standard curve. The molar concentration of libraries was determined by using the Delta R–baseline-corrected raw fluorescence calculated with Aria DX Real-Time PCR Software (Agilent Technologies, Santa Clara, CA, USA). Barcoded libraries (up to 4-Chef runs corresponding to 32 libraries) were super-pooled in equimolar concentration using the strategies suggested for combining libraries prepared with different panels for equal coverage in order to obtain the final molarity of 40 pM each.

### 4.3. Chip Loading and Sequencing

The Ion 510 Chip loading was carried out using the Ion 510, 520, and 530 Kit on the Ion Chef System (Thermo Fisher Scientific, Waltham, MA, USA) following manufacturer instructions. The high throughput sequencing runs were carried out on the Ion Gene Studio S5 system (Thermo Fisher Scientific, Waltham, MA, USA). The run planned in the S5 Torrent Suite (v. 5.12.2) had the following parameters: analysis parameters, default; reference library, hg19; target regions, NBS_LSDs panel BED file; read length, 200 bp; flows, 550; base calibration mode, default. The plugins used were: coverage Analysis, IonReporter Uploader, and Variant Caller (default settings).

### 4.4. Data Analysis

Read mapping was performed automatically in Torrent Suite (v. 5.12.2) by using the variant Caller plugin (v5.12.0.4) with default settings (germline_low_stringency). Called variants were automatically uploaded on Ion Reporter (Thermo Fisher Scientific, Waltham, MA, USA). CNVs performance was not assessed. The pipeline analysis for variants filtering was based on multiple steps in the following order: coverage min 30×, *p*-value < 0.01, ClinVar ≠ Benign or Likely Benign, MAF < 0.001, Frequency 40–60% for heterozygous variants and >85% for homozygous variants, variants effects ≠ synonymous, include intronic variants if the distance from exon is <15 bp. Comparison of Torrent Variant Caller (TVC) variants with their respective truth sets from Coriell biobank was performed post-analysis. True positives (TPs), true negatives (TNs), false positive (FPs), and false negative (FNs) variant calls were defined by considering available data from the single causative gene in the Coriell repository. True positives (TPs) were defined as variants both detected by our filtering pipeline as well as expected from the Coriell collected data. True negatives (TNs) were considered variants neither detected by our pipeline nor expected from repository data. False positives (FPs) were variants detected by our pipeline but not expected from data. False negatives (FNs) were variants expected from the Coriell data but missed by our pipeline. Accuracy was calculated as follows: (TP + TN)/(TP + FP + TN + FN); sensitivity was calculated as follows: TP/(TP + FN); and specificity was calculated as follows: TN/(TN + FP).

## 5. Conclusions

Targeted sequencing represents an appealing approach to improve routine diagnostic strategy, given its low sequencing costs and short sequencing time; however, preliminary analysis to ensure primer pairs amplification efficacy and a good amplicons coverage need to be performed. Each laboratory interested in LSDs neonatal screening should invest in a diagnostic flowchart including both primary biochemical assays and the appropriate molecular genetic tools to address the clinical suspicion. We believe that the broad adoption of NGS panels, such as the one described here, into NBS may increase the yield of LSDs diagnostic process, producing a significant reduction in delayed diagnostic response with beneficial results in treatment outcome.

## Figures and Tables

**Table 1:** Detected and missed pathogenic variants in analyzed samples and new findings.

LSDs Disease	Sample	Ref	Observed Allele	Type	Genes	Hom/Het	Coding	Amino Acid Change	Variant Effect	ClinVar
Gaucher	NA00372	T	C	SNV	*GBA*	Het	c.1226A>G	p.Asn409Ser	missense	CIP
		G	GC	INDEL	*GBA*	Het	c.84_85insG	p.Leu29 AlafsTer18	frameshift insertion	P
		**A**	**G**	**SNV**	***SMPD1***	**Het**	**c.56A>G**	**p.Gln19Arg**	**missense**	**US**
		**G**	**A**	**SNV**	***GAA***	**Het**	**c.2561G>A**	**p.Arg854Gln**	**missense**	**CIP**
	NA00877	A	G	SNV	*GBA*	Hom	c.1448T>C	p.Leu483Pro	missense	CIP
	NA10870	T	C	SNV	*GBA*	Hom	c.1226A>G	p.Asn409Ser	missense	CIP
	NA10874	T	C	SNV	*GBA*	Het	c.1226A>G	p.Asn409Ser	missense	
		not detected [heterozygous 476G>A, Arg120Gln (R120Q)]								
Fabry	NA00107	not detected [hemizygous c.485G>A, Trp162Ter (W162X)]								
	NA00636	ACTT	A	INDEL	*GLA*	Hom	c.177+6063_177+ 6065delCTT, c.1212_1214delAAG	p.Arg404del	non frameshift deletion	P
	NA04391	T	C	SNV	*GLA*	Hom	c.177+7120T>C, c.644A>G	p.Asn215Ser	missense	P
Pompe	NA00244	T	C	SNV	*GAA*	Het	c.953T>C	p.Met318Thr	missense	CIP
		C	T	SNV	*GAA*	Het	c.2560C>T	p.Arg854Ter	nonsense	P
	NA01935	C	A	SNV	*GAA*	Het	c.1935C>A	p.Asp645Glu	missense	P
		C	T	SNV	*GAA*	Het	c.2560C>T	p.Arg854Ter	nonsense	P
	NA14108	T	G	SNV	*GAA*	Het	c.-32-13T>G	p.?	unknown	P
		CT	C	INDEL	*GAA*	Het	c.525delT	p.Glu176 ArgfsTer45	frameshift deletion	P
Niemann Pick A-B	NA00112	T	C	SNV	*SMPD1*	Hom	c.911T>C	p.Leu304Pro	missense	P
	NA13205	TC	T	INDEL	*SMPD1*	Het	c.996delC	p.Phe333 SerfsTer52	frameshift deletion	P
		**A**	**C**	**SNV**	***SMPD1***	**Het**	**c.1172A>C**	**p.Asn391Thr**	**missense**	**n.a.**
	NA16193	G	T	SNV	*SMPD1*	Het	c.1493G>T, c.*1330C>A	p.Arg498Leu	missense	P
		TGCC	T	INDEL	*SMPD1*	Het	c.1829_1831delGCC, c.*998GGCA>A	p.Arg610del	non frameshift deletion	n.a.
MPSI	NA00798	**A**	**G**	**SNV**	***GBA***	**Het**	**c.1448T>C**	**p.Leu483Pro**	**missense**	**CIP**
		G	A	SNV	*IDUA*	Hom	c.1205G>A	p.Trp402Ter	nonsense	P
	NA01256	G	A	SNV	*IDUA*	Het	c.590-7G>A	p.?	unknown	P
		G	A	SNV	*IDUA*	Het	c.1205G>A	p.Trp402Ter	nonsense	P

SNV = Single Nucleotide polymorphism; P = Pathogenic; US = Uncertain Significance; CIP = Conflicting Interpretation of Pathogenicity. New observed findings are reported in bold.

**Table 2:** Cost per sample (update June 2021) of consumables for semi-automatic sequencing with NBS_LSDs panel.

	Number of Processed Samples	Price (Euro)	Cost/Sample (Euro)
QIAmp DNA Micro Kit	50	215	4.3
TaqMan™ RNase P Detection Reagents Kit (100 rxn)	33 *	367	11.1
TaqMan™ Universal PCR Master Mix (500 rxn)	166 *	584	3.5
Made-To-Order Ion AmpliSeq panel NBS_LSDs 2X (IAD199968_236)	1000	1422,40	1.4
Ion AmpliSeq™ Kit for Chef DL8	32	4660	145.6
Ion Library TaqMan^®^ Quantitation Kit (250 rxn)	83 *	1546	18.6
Ion 510™ & Ion 520™ & Ion 530™ Kit–Chef (2 sequencing runs per initialization, 8 loaded chips)	256 (32/chip)	3600,00	14.5
Ion 510™ Chip Kit (8-Pack)	256 (32/chip)	4665	18.2
Total cost per sample			217.30

* samples are considered as processed in triplicate.

## Data Availability

Details of the reference samples selected for the present validation can be found at https://www.coriell.org/.

## Supplementary Materials

The following are available online at https://www.mdpi.com/article/10.3390/ijms221810064/s1.
